# Generation of a variety of stable Influenza A reporter viruses by genetic engineering of the NS gene segment

**DOI:** 10.1038/srep11346

**Published:** 2015-06-12

**Authors:** Peter Reuther, Kristina Göpfert, Alexandra H. Dudek, Monika Heiner, Susanne Herold, Martin Schwemmle

**Affiliations:** 1 Institute for Virology, University of Freiburg, 79104 Freiburg, Germany; 2 Faculty of Biology, University of Freiburg, 79104 Freiburg, Germany; 3 Spemann Graduate School of Biology and Medicine (SGBM), University of Freiburg, 79104 Freiburg, Germany; 4 Department of Internal Medicine (Pulmonology), University of Giessen and Marburg Lung Center, German Center for Lung Research, D-35392 Giessen, Germany

## Abstract

Influenza A viruses (IAV) pose a constant threat to the human population and therefore a better understanding of their fundamental biology and identification of novel therapeutics is of upmost importance. Various reporter-encoding IAV were generated to achieve these goals, however, one recurring difficulty was the genetic instability especially of larger reporter genes. We employed the viral NS segment coding for the non-structural protein 1 (NS1) and nuclear export protein (NEP) for stable expression of diverse reporter proteins. This was achieved by converting the NS segment into a single open reading frame (ORF) coding for NS1, the respective reporter and NEP. To allow expression of individual proteins, the reporter genes were flanked by two porcine Teschovirus-1 2A peptide (PTV-1 2A)-coding sequences. The resulting viruses encoding luciferases, fluorescent proteins or a Cre recombinase are characterized by a high genetic stability *in vitro* and in mice and can be readily employed for antiviral compound screenings, visualization of infected cells or cells that survived acute infection.

Influenza A viruses (IAV) cause severe respiratory illness in humans and account for 250,000–500,000 annual deaths worldwide^1^. Especially zoonotic transmission of IAV from avian reservoirs poses a constant threat to the human population^2^, as exemplified recently by several fatal human cases upon H5N1 or H7N9 infections^3^,^4^. Albeit infrequently, avian IAV can also establish new, aerosol-transmissible virus lineages in humans, resulting in devastating pandemics with high morbidity and mortality^2^. The development of effective countermeasures, such as vaccines or therapeutics has been complicated by the ability of the viruses to rapidly mutate antigenic determinants or antiviral target structures^5^,^6^,^7^. Improved vaccine approaches, identification of new antivirals and in general a better understanding of the fundamental biology of IAV infection is required to efficiently antagonize these human pathogens in future^8^,^9^.

To achieve these tasks, a number of IAV encoding luciferases^10^,^11^,^12^,^13^,^14^,^15^ or fluorescent proteins^15^,^16^,^17^,^18^ have been recently generated. The genome of IAV consists of 8 RNA segments with negative polarity^19^. These segments are numbered according to their size ranging from 2.3 kb (segment 1) to 0.9 kb (segment 8). Segments 1–3 encoding the polymerase subunits PB2, PB1 and PA respectively, segment 6 coding for the neuraminidase (NA) and segment 8 (NS) encoding both the non-structural protein 1 (NS1) and the nuclear export protein (NEP) have been shown to tolerate insertion of foreign genes, including that for the green fluorescence protein (GFP) with a length of around 0.7 kb^11^,^12^,^13^,^14^,^15^,^16^,^17^,^18^. However, the integration of a reporter gene of this size represents a substantial increase of the overall segment length and is accompanied by substantial attenuation of viral replication^11^,^14^,^15^,^16^. As a consequence, especially viruses comprising the GFP gene were shown to lose reporter activity after passage in cell culture or mice^15^,^16^,^20^, which could render them unfavorable for many experimental approaches, such as multicycle growth experiments, long-term infection of model organisms or transmission studies.

The NS-segment comprises two overlapping ORFs coding for NS1 and NEP. While NS1 is translated from an intron-containing mRNA transcript, the NEP mRNA is generated by exploitation of the cellular splicing machinery (Fig. 1A)^21^. Manicassamy *et al.* were the first to utilize the NS-segment as vector for transgene expression in influenza A virus infected cells^16^. There, an NS1-GFP fusion protein and NEP are encoded by two non-overlapping genes, which are separated by the porcine Teschovirus-1 2A peptide (PTV-1 2A) coding sequence. PTV-1 2A mediates co-translational separation of NS1-GFP and NEP by a mechanism termed “stop-carry on” recoding^22^. The NS1-GFP expressing virus was found to be attenuated in cell culture and mice and it was proposed that this attenuation is based on an imbalance of NS1 and NEP protein levels, as the coordinated expression of both proteins is no longer regulated by splicing^23^. In contrast to the NS1-GFP-expressing virus, however, we could recently show that processing of NS1 and NEP from two non-overlapping genes separated by PTV-1 2A does not result in detectable impairment of viral replication in cell culture and mice^24^. Based on this favorable property of PTV-1 2A, we engineered influenza A viruses harboring an NS segment encoding reporter genes flanked by two genetically distinct PTV-1 2A-encoding sequences. These viruses are genetically stable in cell culture and mice and express a variety of luminescent and fluorescent reporters as well as a Cre recombinase.

## Results

### PTV-1 2A-mediated co-translational separation of NS1 and NEP does not interfere with viral replication

**Figure 1:**
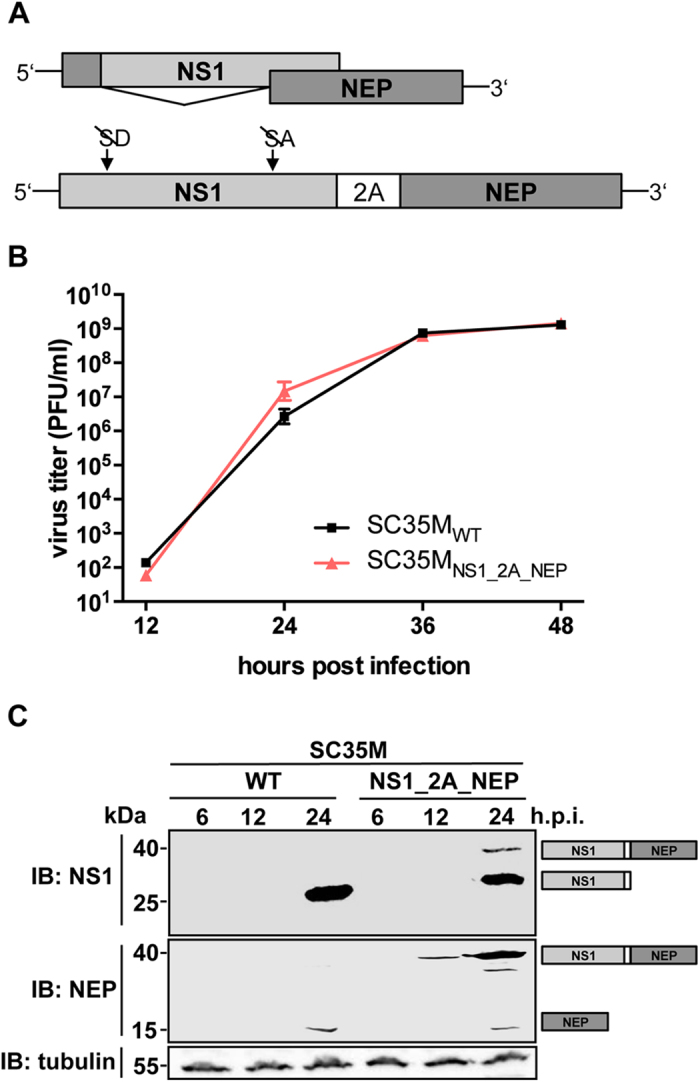
PTV-1 2A mediated processing of NS1 and NEP does not compromise viral replication. **(A)** Schematic representation of the wild type NS segment and the NS1_2A_NEP segment. In the latter mutation of splice donor (SD) and acceptor site (SA) prevents splicing. NS1 and NEP are co-translationally separated by the recoding activity of the 2A peptide of porcine teschovirus 1 (PTV-1). Note that the sizes of the respective genes are not in scale **(B)** Viral growth of SC35M_WT_ and SC35M_NS1_2A_NEP_ in MDCK-II cells. Viral titers in the supernatant of cells infected at an MOI of 0.001 were determined by plaque assay at the indicated time points post infection. Error bars represent standard error of the mean from three independent experiments. **(C)** Determination of the expression levels of NS1 and NEP in A549 cells infected with SC35M_WT_ or SC35M_NS1_2A_NEP_ at an MOI of 0.1 at the indicated time points by Western blot analysis. The immunoblot (IB) was carried out with NS1- and NEP-specific antibodies. Detection of tubulin served as loading control. Proteins corresponding to the size of the detected bands are indicated

To confirm that the NS-segment of the mouse-adapted IAV A/SC35M (H7N7)^25^ allows satisfying co-translational separation of NS1 and NEP, we generated a pHW2000 based^26^ rescue plasmid (NS1_2A_NEP) that encodes NS1 and NEP in a single ORF (Fig. 1A). Here, the splice donor and acceptor sites of the NS1 gene were silenced by site-directed mutagenesis without affecting the amino acid sequence. A PTV-1 2A-coding sequence was introduced to mediate co-translational separation of NS1 and NEP. As expected from our previous work^24^, a recombinant virus carrying this modified NS-segment (SC35M_NS1_2A_NEP_) replicates as efficiently as wild type SC35M (SC35M_WT_) in mammalian MDCK-II cells (Fig. 1B). To analyze whether this modified NS-segment causes an altered NS1:NEP protein ratio during infection, we determined the levels of these proteins in the lysates of MDCK-II cells infected with SC35M_WT_ or SC35M_NS1_2A_NEP_ at a multiplicity of infection (MOI) of 0.1. As shown in Fig. 1C, NS1 (26 kDa) and NEP (14.5 kDa) protein levels were comparable between the two viruses 24 hours post infection (h.p.i.). However, we also detected a 40 kDa band corresponding to an NS1-2A-NEP polyprotein with both an NS1- and an NEP-specific antibody in the cell lysate of SC35M_NS1_2A_NEP_-infected cells, indicating that the 2A-mediated “stop-carry on” recoding is not completely efficient.

### Fusion of a GFP to the nuclear export protein (NEP) results in compromised viral growth

**Figure 2:**
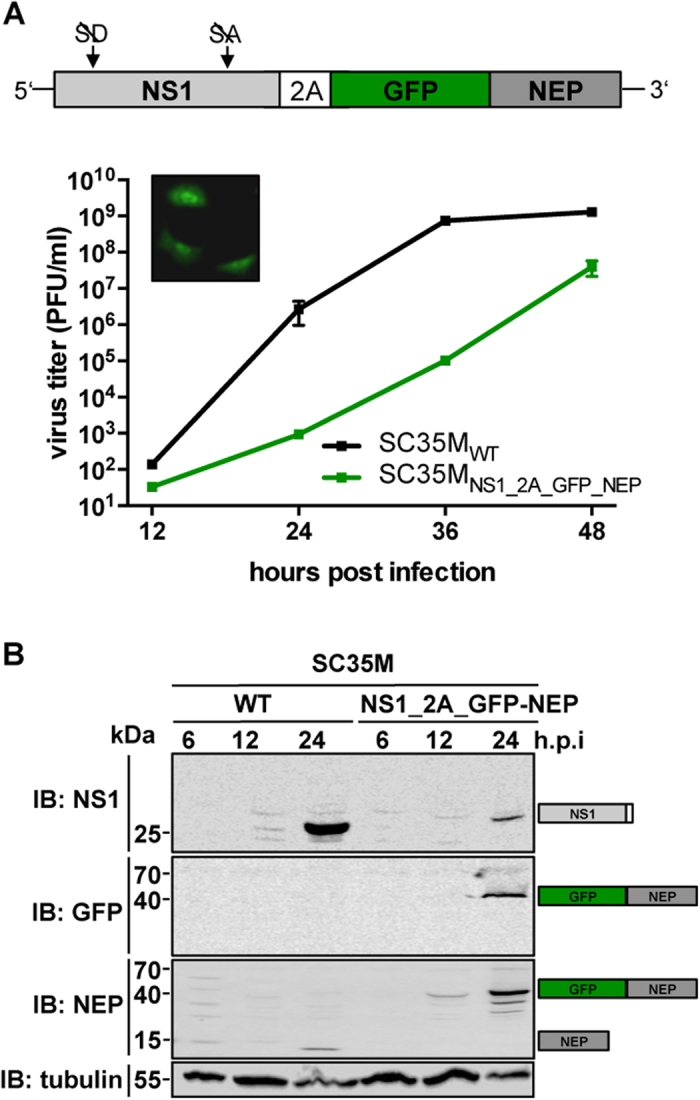
Fusion of GFP to NEP results in substantial attenuation. **(A)** Viral growth of SC35M_WT_ and SC35M_NS1_2A_GFP-NEP_ in MDCK-II cells. Viral titers in the supernatant of cells infected at an MOI of 0.001 were determined by plaque assay at the indicated time points. Error bars represent standard error of the mean from three independent experiments. The cartoon illustrates the design of the NS1_2A_GFP-NEP segment. The confocal microscopy image in the upper left corner shows GFP-positive A549 cells infected at an MOI of 5 with SC35M_NS1_2A_GFP-NEP_ 6 hours post infection. **(B)** Immunoblot (IB) analysis of expression levels of NS1, NEP and GFP in A549 cells infected with SC35M_WT_ or SC35M_NS1_2A_GFP-NEP_ at an MOI of 0.1 at the indicated time points. Proteins corresponding to the size of the detected bands are indicated.

Based on recent observation that N-terminally GFP-tagged NEP (GFP-NEP) retains its nuclear export and polymerase co-factor function *in vitro*^27^,^28^, we reasoned that reporter genes might be introduced into the viral genome as NEP-fusion constructs. To demonstrate this, we generated a pHW2000-based NS segment rescue plasmid coding for NS1, 2A and a GFP-NEP fusion protein (Fig. 2A). Using this plasmid, we could indeed successfully generate a recombinant GFP-encoding virus (SC35M_NS1_2A_GFP-NEP_). SC35M_NS1_2A_GFP-NEP_ is characterized by severe attenuation in MDCK-II cells, which was most obvious at 24 and 36 h.p.i., when viral titers in the cell supernatant were reduced by several log_10_ relative to cells infected with SC35M_WT_ (Fig. 2A). In contrast to SC35M_NS1_2A_NEP_ (Fig. 1C), we observed highly elevated levels of NEP in lysates of cells infected with SC35M_NS1_2A_GFP-NEP_ (Fig. 2B). This is consistent with earlier observations that fusion of GFP increases the stability of NEP^27^ and that higher levels of NEP impair viral growth^23^. Although there is an increasing body of evidence that NEP is a multifunctional protein crucial for vRNP nuclear export, polymerase activity and viral budding^29^,^30^, little is known about the spatiotemporal regulation of these diverse functions. As we hardly detected any NS1-2A-GFP-NEP polyprotein in the lysate of SC35M_NS1_2A_GFP-NEP_-infected cells (Fig. 2B), this virus might serve as a valuable tool for the visualization of changes in the subcellular localization of NEP or its interaction with host factors in the course of an infection.

### Introduction of a second PTV-1 2A peptide permits the generation of stable reporter viruses

**Figure 3:**
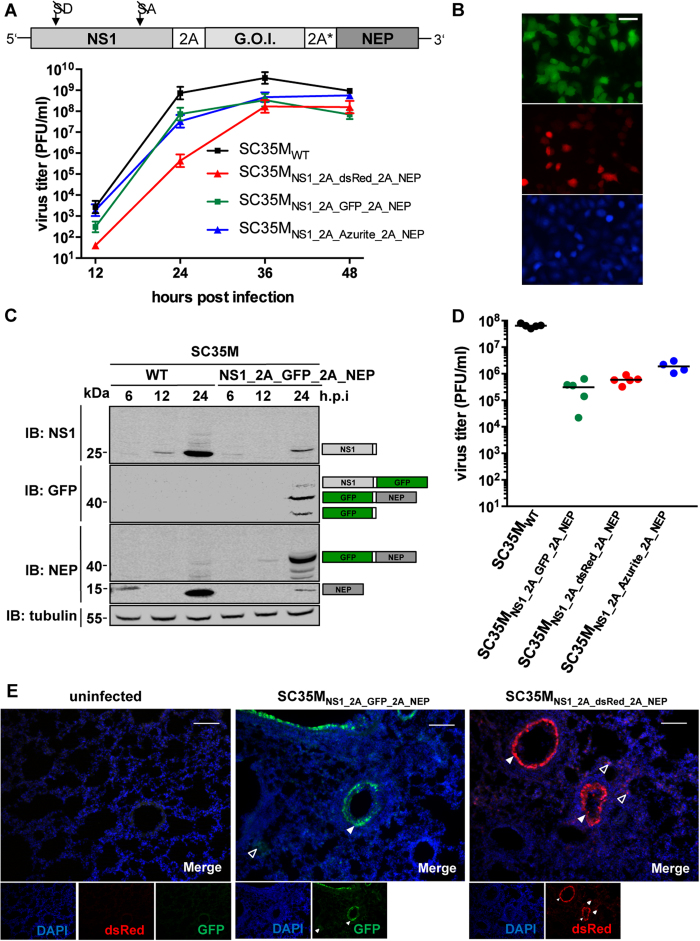
Insertion of a second PTV-1 2A peptide enhances replication of fluorescent reporter viruses. **(A)** Viral growth of SC35M_WT_ and the indicated fluorescent reporter viruses in MDCK-II cells. Viral titers in the supernatant of cells infected at an MOI of 0.001 were determined by plaque assay at the indicated time points post infection. Error bars represent standard error of the mean from three independent experiments. The cartoon is a schematic representation of the modified NS segment. A genetically altered sequence encoding a second PTV-1 2A peptide (2A*) was inserted between the gene of interest (G.O.I.) and NEP. **(B)** Fluorescent live cell imaging of A549 cells infected at an MOI of 1 with SC35M_NS1_2A_GFP_2A_NEP_ (upper panel), SC35M_NS1_2A_dsRed_2A_NEP_ (middle panel) or SC35M_NS1_2A_Azurite_2A_NEP_. Scale bar represents 100 μm. **(C)** Immunoblot (IB) analysis of expression levels of NS1, NEP and GFP in A549 cells infected with SC35M_WT_ or SC35M_NS1_2A_GFP_2A_NEP_ at an MOI of 0.1 at the indicated time points post infection. Proteins corresponding to the size of the detected bands are indicated. **(D)** Determination of lung titers from 6–8 week old female BALB/c mice (n = 4 or 5) infected intranasally with 1,000 PFU of the indicated viruses 48 hours post infection. **(E)** Fluorescent imaging of lung sections from BALB/c mice infected intranasally with 10,000 PFU of the indicated viruses or mock infection respectively. Lungs were collected 48 hours post infection. Filled arrowheads indicate infected cells within the epithelial layers of larger airways, whereas transparent arrowheads indicate infected cells of smaller airways. Scale bars represent 100 μm.

The fusion of GFP to NEP substantially compromised viral growth (Fig. 2A). Since this represents a major drawback for many experimental approaches, we set out to generate rescue plasmids allowing the expression of a gene of interest without its fusion to NS1 or NEP. To achieve this, we introduced a second PTV-1 2A-coding sequence between the transgene and NEP (Fig. 3A). This sequence was genetically modified to the highest possible extent to prevent homologous recombination with the non-modified PTV-1 2A nucleotide sequence (see Material and Methods). Using this approach, we were able to rescue viruses encoding three different fluorescent reporter genes: GFP (SC35M_NS1_2A_GFP_2A_NEP_), its blue fluorescent derivate Azurite (SC35M_NS1_2A_Azurite_2A_NEP_)^31^ and the red fluorescent protein dsRed (SC35M_NS1_2A_dsRed_2A_NEP_). All reporter viruses replicated to similar high viral titers of 10^8^ PFU/ml 36 h.p.i., which represents an attenuation of approximately one log_10_ relative to SC35M_WT_ (Fig. 3A). Replication of SC35M_NS1_2A_dsRed_2A_NEP_ was particularly delayed, as indicated by the low viral titer 24 hours post infection (Fig. 3A). As expected, infection of A549 cells with the reporter viruses resulted in well detectable fluorescence signals performing live cell imaging microscopy (Fig. 3B). To analyze the genetic stability of the modified NS segments, viruses were passaged four times in A549 cells and the proportion of reporter-expressing infectious viral particles was determined by plaque assay and subsequent fluorescent microscopy. As shown in table 1, all viruses retained their reporter gene after passaging in human cells, indicating a favorable stability of the reporter gene at least over 4 rounds of passaging. To analyze the recoding efficiency of the two PTV-1 2A peptides, NS1, GFP and NEP protein levels were determined in lysates of cells infected with SC35M_NS1_2A_GFP_2A_NEP_ (Fig. 3C). Besides detection of the three individual proteins, we observed with both GFP- and NEP-specific antibodies a protein band of approximately 40 kDa corresponding to a GFP-2A-NEP fusion protein (Fig. 3C). A faint signal, which is visible above this GFP-2A-NEP band in the GFP-immunoblot might indicate the presence of low levels of an NS1-2A-GFP construct. Interestingly, we could not visualize a high molecular weight band corresponding to an NS1-2A-GFP-2A-NEP polyprotein. Consistently, we observed high levels of unprocessed reporter proteins fused to NEP in the lysates of cells infected with SC35M_NS1_2A_dsRed_2A_NEP_ and SC35M_NS1_2A_Azurite_2A_NEP_ (Fig. S1). To further characterize the fluorescent reporter viruses *in vivo*, BALB/c mice were infected intranasally with 1,000 PFU of the respective viruses and viral lung titers were determined 48 h.p.i. All reporter viruses replicated to significant titers between 5 × 10^5^ and 5 × 10^6^ PFU/lung (Fig. 3D). This also included SC35M_NS1_2A_dsRed_2A_NEP_, which showed delayed replication properties in cell culture compared to both SC35M_NS1_2A_GFP_2A_NEP_ and SC35M_NS1_2A_Azurite_2A_NEP_ (Fig. 3A). However, viral lung titers of all reporter viruses were substantially lower than the titers observed after infection of BALB/c mice with SC35M_WT._ Accordingly, the LD_50_ of the fluorescent reporter viruses is increased compared to SC35M_WT_^32^ (Table 2). To monitor the genetic stability of the three reporter viruses *in vivo*, plaque assays were performed on the lung homogenates of the infected BALB/c mice and screened for reporter expression by fluorescent microscopy. This revealed that in all analyzed plaques (100/reporter virus) reporter expression was maintained, again demonstrating the high genetic stability of the here presented viruses (Table 1). To visualize spread of the reporter viruses in lungs of infected animals, BALB/c mice were infected intranasally with 10,000 PFU of SC35M_NS1_2A_GFP_2A_NEP_ or SC35M_NS1_2A_dsRed_2A_NEP_. 48 hours post infection lungs were collected and subjected to microscopic analysis. Virus replication, as monitored by green or red fluorescence, could be observed in the epithelial layer of the bronchiolar tube (Fig. 3E, filled arrow heads) as well a in the distal lung tissues (Fig. 3E, transparent arrow heads). Taking together, these data indicate that our fluorescent reporter viruses are genetically stable and allow tracing of different virus-infected lung cells in mice.

### Generation of luciferase-encoding viruses for screening approaches

**Figure 4:**
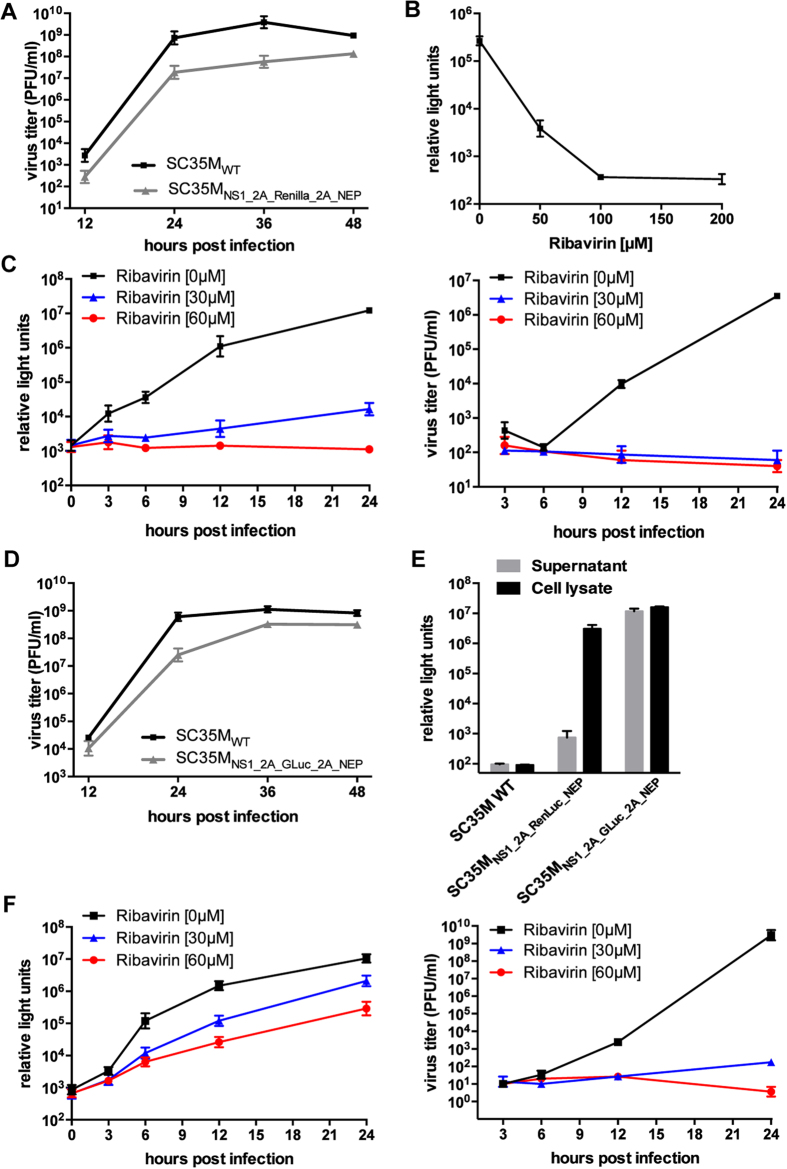
Luciferase encoding viruses as tools for antiviral compound screenings. **(A)** Viral growth of SC35M_WT_ and SC35M_NS1_2A_RenLuc_2A_NEP_ in MDCK-II cells. Viral titers of cells infected at an MOI of 0.001 at the indicated time points post infection were determined by plaque assay. Error bars indicate standard error of the mean from three independent experiments. **(B)** Determination of luciferase activity in the lysates of MDCK-II cells infected with an MOI of 0.01 of SC35M_NS1_2A_RenLuc_2A_NEP_ and treated with the indicated concentration of ribavirin 24 hours post infection. Error bars indicate standard deviation from three independent experiments. **(C)** Luciferase activity (left panel) determined from the lysate or viral titers determined from the supernatant (right panel) of MDCK-II cells infected with an MOI of 0.01 of SC35M_NS1_2A_RenLuc_2A_NEP_ and treated with the indicated concentrations of ribavirin. Error bars indicate standard deviation from three independent experiments. **(D)** Viral growth of SC35M_WT_ and SC35M_NS1_2A_GLuc_2A_NEP_ in MDCK-II cells were determined as described in (A) **(E)** Luciferase activity in the supernatant and the lysate of MDCK-II cells infected with the indicated viruses (MOI of 0.01) 24 hours post infection. Error bars indicate standard deviation from three independent experiments. **(F)** Luciferase activity (left panel) and virus titers (right panel) in the supernatant of MDCK-II cells infected with SC35M_NS1_2A_GLuc_2A_NEP_ at an MOI of 0.01 of SC35M_NS1_2A_GLuc_2A_NEP_ and treated with 0, 30 or 60 μM ribavirin at the indicated time points post infection. Error bars indicate standard deviation from three independent experiments.

Luciferases have been proven to be valuable tools in screening approaches for the identification of novel antiviral substances or host factors^33^,^34^,^35^,^36^. To generate a stable, luciferase-encoding virus, the gene for the Renilla luciferase (RenLuc) (~0.9 kB) was introduced into the NS-segment. MDCK-II cells infected with the resulting virus (SC35M_NS1_2A_RenLuc_2A_NEP_) at an MOI of 0.001 released 10^8^ PFU/ml into the cell supernatant after 48 hours (Fig. 4A). However, compared to SC35M_WT_-infected cultures, SC35M_NS1_2A_RenLuc_2A_NEP_ revealed impaired viral growth. To prove stable expression of luciferase, SC35M_NS1_2A_RenLuc_2A_NEP_ was passaged 4 times in human A549 cells. Reporter analysis of ten plaque-purified viruses revealed no loss of luciferase activity (Tab. 1), indicating stable expression of the RenLuc gene. To analyze whether SC35M_NS1_2A_RenLuc_2A_NEP_ could be employed for antiviral compound screenings, we treated infected MDCK-II cells with increasing concentrations of the viral polymerase inhibitor ribavirin and determined luciferase activity 24 hours post infection. As shown in Fig. 4B, RenLuc activity decreased in a dose-dependent manner reaching a baseline at approximately 100 μM of ribavirin. The classic approach to analyze the antiviral activity in cell culture is the determination of viral titers. There, effects at an early stage within the first 6 hours of infection, prior to efficient particle formation, cannot be visualized. As the expression of virus-encoded luciferase is dependent on viral RNA synthesis and not particle release, we reasoned that SC35M_NS1_2A_RenLuc_2A_NEP_ would allow quantification of antiviral activity of the viral polymerase inhibitor ribavirin at early time points post infection. To show this, SC35M_NS1_2A_RenLuc_2A_NEP_-infected MDCK-II cells were cultured in the presence of 0, 30 or 60 μM of ribavirin and analyzed at different time points post infection for viral titers in the supernatant and luciferase activity in the cell lysate (Fig. 4C). Already at 3 h.p.i., we observed a substantial dose dependent difference in luciferase activity, whereas a first reduction of viral titers was detected at 12 hours post infection. Furthermore, we could not resolve differences in the antiviral efficacy between cells treated with 30 μM or 60 μM ribavirin by determination of viral titers, while this was possible as soon as 6 hours post infection by measuring luciferase activity.

The Gaussia luciferase (GLuc) represents an attractive luminescent reporter for many experimental approaches, not least due to its small size and its secretion from mammalian cells^37^,^38^. Indeed, insertion of the GLuc-coding sequence into the viral NS segment revealed a recombinant virus (SC35M_NS1_2A_GLuc_2A_NEP_), which was only partially attenuated but highly stable in cell culture (Fig. 4D, Tab.1). As expected, the supernatant of MDCK-II cells infected with this virus contained significant levels of secreted GLuc (Fig. 4E). To evaluate whether this extracellular reporter activity could be exploited as a measure for viral replication, we infected MDCK-II cells in the presence of ribavirin and determined luciferase activity and virus titers from the supernatant at different time points post infection. As anticipated, luciferase activity differed dependent on the concentration of ribavirin (Fig. 4F). However, especially at 24 hours post infection, differences in luciferase activity were not proportional to differences in virus titers determined from the same supernatants, most probably due to extracellular accumulation of the highly stable GLuc^38^. As for the fluorescent reporter viruses, we detected high levels of unprocessed, luciferase-NEP fusion proteins in lysates of cells infected with SC35M_NS1_2A_GLuc_2A_NEP_ and SC35M_NS1_2A_RenLuc_2A_NEP_ (Fig. S1).

### A Cre recombinase-encoding virus allows visualization of cells that are infected or survived acute infection

**Figure 5:**
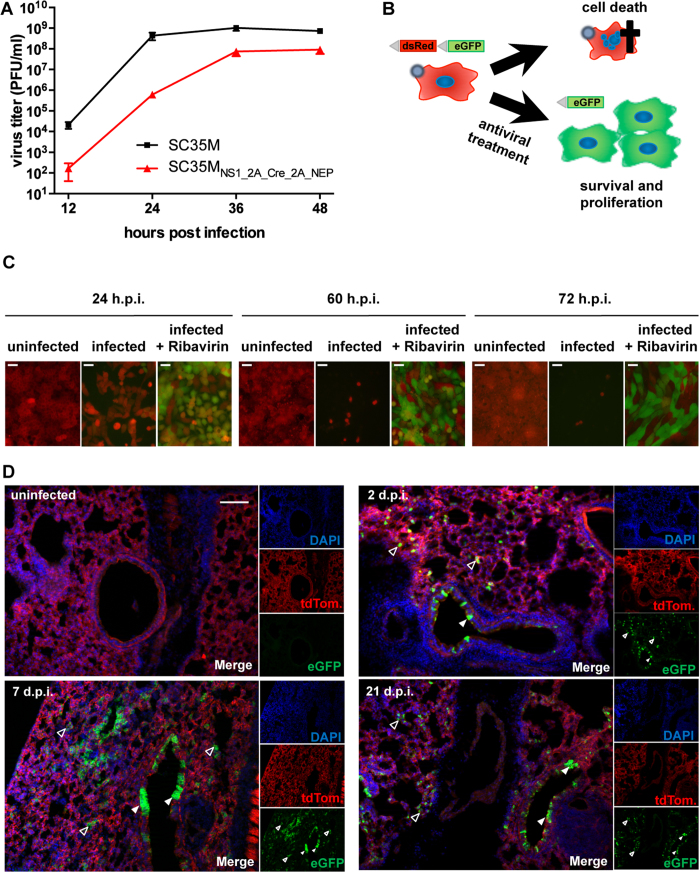
The Cre-recombinase encoding virus induces Cre-Lox recombination *in vitro* and *in vivo*. **(A)** Viral growth of SC35M_WT_ and SC35M_NS1_2A_Cre_2A_NEP_ in MDCK-II cells infected at an MOI of 0.001. Error bars depict standard error of the mean from three independent experiments. **(B)** Cartoon illustrating the experimental procedure to visualize Cre-mediated recombination events in SC35M_NS1_2A_Cre_2A_NEP_-infected cells. Based on the strong cytopathic effect associated with influenza A virus infections, Calu-3 cells harboring a loxP-flanked dsRed upstream of an eGFP gene will undergo cell death after infection with SC35M_NS1_2A_Cre_2A_NEP_ and as a consequence recombined, GFP positive cells cannot be easily identified. However, antiviral treatment with ribavirin 3 hours post infection might inhibit viral replication allowing GFP expression upon Cre-lox recombination. **(C)** Fluorescent live cell imaging of infected Calu-3 cells 24, 60 and 72 h post infection with SC35M_NS1_2A_Cre_2A_NEP_ (MOI of 1) and treatment with ribavirin (100 μM) 3, 24 and 48 hours post infection (h.p.i.). Note the decline of dsRed-positive cells in infected but untreated cultures. Scale bars represent 100 μm. **(D)** Fluorescent imaging of lung sections from rosa^mT/mG^ mice infected intranasally with 2 × 10^6^ PFU of SC35M_NS1_2A_Cre_2A_NEP_. Lungs were collected at the indicated time points post infection. Filled arrowheads point to recombined cells within the epithelial layers of larger airways, transparent arrowheads to those of smaller airways. Scale bar represents 100 μm.

**Figure 6:**
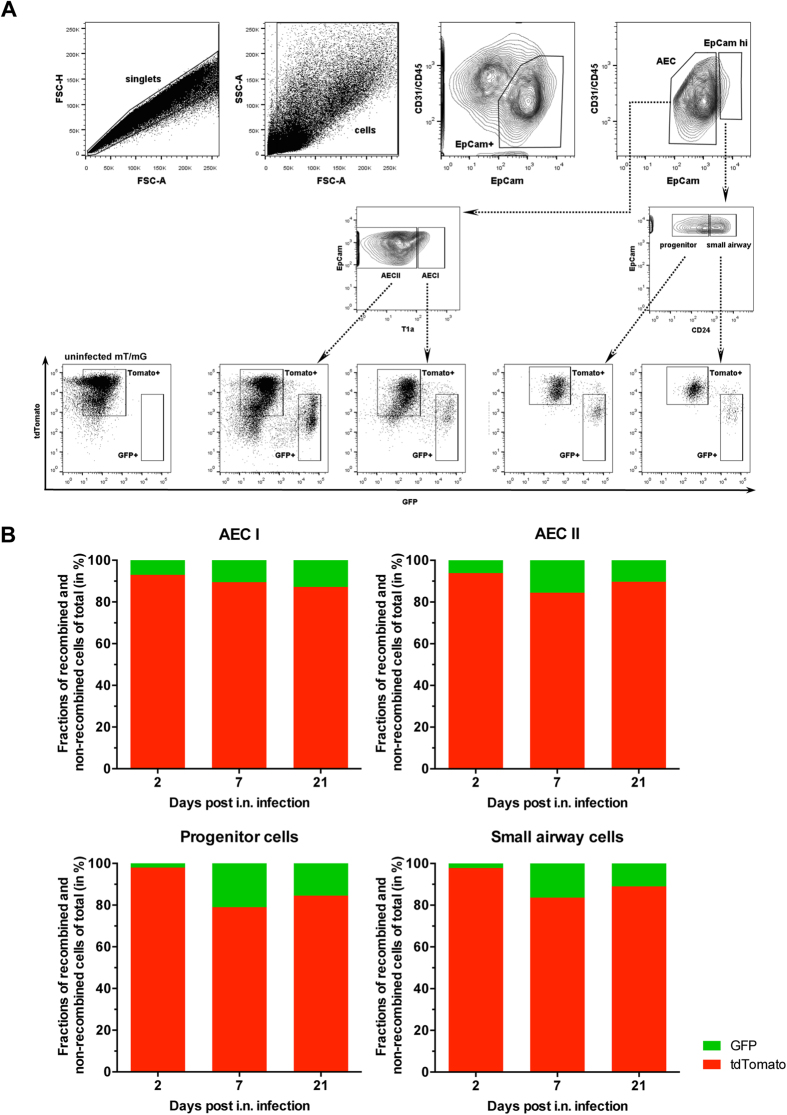
Flow cytometric analysis of murine lungs from SC35M_NS1_2A_Cre_2A_NEP_-infected rosa^mT/mG^ mice 2, 7 and 21 days post infection. Uninfected rosa^mT/mG^ mice served as a control. (A) FACS plots and sequential gating strategy to identify recombined (GFP+) and non-recombined (tdTomato+) cell populations within different compartments of the lung. Day 7 post infection is shown representatively. After doublet and debris exclusion, epithelial cells (CD31^-^CD45^-^EpCam^+^) were distinguished from non-epithelial cells comprising endothelial cells (CD31^+^CD45^-^EpCam^-^), leukocytes (CD31^-^CD45^+^EpCam^-^), and other cells (CD31^-^CD45^-^EpCam^-^). Epithelial cells were subgated as previously described^57^ and were composed of EpCam^low^ alveolar epithelial cells, which constituted of T1α^+^ alveolar epithelial cells type I (AEC I) and T1α^-^ alveolar epithelial cells type II (AEC II). Remaining bronchial EpCam^high^ cells were composed of CD24^high^ small airway epithelial cells (ciliated cells, goblet cells, club cells) and CD24^low^ stem/progenitor cells. FACS plots showing GFP+ and tdTomato+ cell populations within the characterized epithelial cell types are depicted below. (B) Proportions of recombined (GFP+) and non-recombined (tdTomato+) cells within the respective epithelial cell populations analyzed at the indicated time points post infection.

The Cre-Lox recombination is a widely used method to control gene expression in cell culture or animal models^39^,^40^. To generate a genetically stable virus that would allow activation or deactivation of genes specifically in infected cells, we introduced a Cre recombinase gene into the NS segment (SC35M_NS1_2A_Cre_2A_NEP_). Infection of MDCK-II cells revealed a high viral titer of 10^8^ PFU/ml cell culture supernatant 48 h post infection (Fig. 5A). However, SC35M_NS1_2A_Cre_2A_NEP_ showed a delayed viral growth especially at 12 and 24 hours post infection compared to SC35M_WT_ (Fig. 5A). To functionally monitor expression of Cre, we established a human airway derived Calu-3 cell line harboring a loxP-flanked dsRed gene followed by a silenced eGFP gene^41^ (Fig. 5B). Upon Cre-mediated recombination, the dsRed gene is eliminated and cells express eGFP, resulting in a switch from red to green fluorescence and consequently constitutive GFP expression is inherited to progeny cells after proliferation. As IAV suppress host protein synthesis and induce apoptosis^42^,^43^,^44^, we expected that viral infection would complicate identification and analysis of reporter cells upon virus-induced recombination. Indeed, as judged by live cell imaging, infected Calu-3 cells only showed faint green fluorescence 24 hours post infection and were almost completely eliminated at later time points (Fig. 5C, infected). To prevent virus-induced cell death and allow limited viral replication, we treated virus-infected cells with ribavirin (0.1 mM) 3 hours post infection and kept them under treatment to abrogate further replication. We anticipated that this would allow sufficient expression of Cre recombinase and the associated switch to GFP expression as well as prolonged cell survival (Fig. 5B). Indeed, increasing number of GFP-positive Calu-3 reporter cells were observed in a time dependent manner upon infection at an MOI of 1 with SC35M_NS1_2A_Cre_2A_NEP_ and subsequent ribavirin treatment (Fig. 5C, infected + ribavirin). As we could show in cell culture that SC35M_NS1_2A_Cre_2A_NEP_ allows the visualization of cells that survived IAV infection, we hypothesized that we could also trace and identify such cells *in vivo* using a suitable mouse model. For this reason, rosa^mT/mG^ mice^45^ were infected intranasally with a sublethal dose of SC35M_NS1_2A_Cre_2A_NEP_. These transgenic animals comprise a loxP-flanked tdTomato ORF upstream of an eGFP gene integrated into the Rosa26 locus. Analogous to the earlier described cell culture system, Cre-mediated recombination results in a switch from red to green fluorescence in virtually all cell types. Infected animals were sacrificed at 2, 7 or 21 days post infection to collect their lungs for microscopic examination. Two and 7 days after infection, green fluorescent cells could be detected within the epithelial layer of bronchioles (Fig. 5D, filled arrow heads) as well as in distal lung tissues (Fig. 5D, transparent arrow heads). A similar distribution of GFP-positive cells was observed 21 days post infection, a time point where infection was already cleared (data not shown), indicating that diverse populations of epithelial cells survived the virus infection. Interestingly, while 2 days post infection only individual cells in alveolar tissues showed green fluorescence, clusters of adjacent GFP-positive cells could be visualized already 7 days p.i., which might derive from progenitor cells that survived acute infection. To reveal the identity of the GFP-positive cells found in the distal lung compartment, we subjected lung cells from rosa^mT/mG^ mice infected with SC35M_NS1_2A_Cre_2A_NEP_ to flow cytometric analysis at days 2, 7 and 21 post infection. We could identify recombined, GFP-positive cells within lung epithelial cell populations (EpCam^+^), including alveolar epithelial cells type 1 (AEC I, EpCam^low^T1α^+^), alveolar epithelial cells type 2 (AEC II, EpCam^low^T1α^–^), stem and/or progenitor cells (EpCam^high^CD24^low^) and small airway epithelial cells (EpCam^high^CD24^high^) (Fig. 6 A and B). Of note, all infectious viruses isolated from the lungs of infected animals by plaque purification 2 days post infection encoded a functional Cre recombinase as judged by GFP expression in the Calu-3 reporter cells, highlighting the genetic stability of SC35M_NS1_2A_Cre_2A_NEP_ in mice (Table 1). Taken together, this reporter virus in combination with flow cytometric analysis represents a powerful tool to identify and quantify acutely infected lung cells as well as cells that survived acute infection.

### Stable introduction of transgenes into the PB2-segment of SC35M is at the cost of viral replication capacity

**Figure 7:**
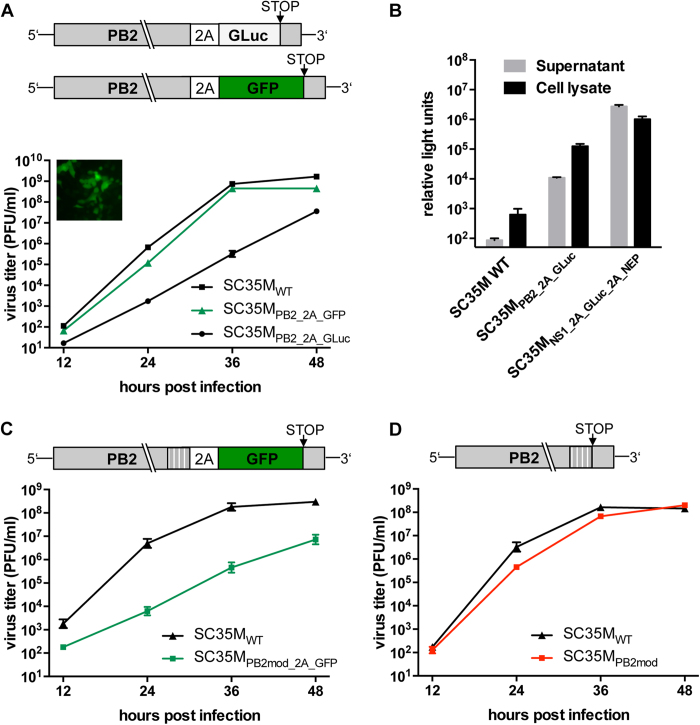
Stable introduction of a reporter gene into the PB2-segements results in severe attenuation. **(A)** Viral growth of SC35M_WT_ and the indicated reporter viruses in MDCK-II cells. Virus titers in the supernatant of MDCK-II cells infected at an MOI of 0.001 were determined by plaque assay at the indicated time points. Error bars indicate standard error of the mean from three independent experiments. The cartoon schematically represents the modified PB2 segments. The fluorescent live cell image in the upper left corner depicts the GFP-positive A549 cells infected at an MOI of 1 of SC35M_PB2_2A_GFP_ 24 hours post infection. **(B)** Luciferase activity in the supernatant and the lysate of MDCK-II cells infected the indicated viruses at an MOI of 0.01, 24 hours post infection. Error bars indicate standard deviation from three independent experiments. **(C and D)** Viral growth of SC35M_WT_, SC35M_PB2mod_2A_GFP_ (C) and SC35M_PB2mod_ (D) was determined as described above. The cartoons schematically represent the modified PB2 segments. Shaded boxes indicate silent mutations introduced into the last 129 nucleotides of the PB2 ORF.

As for the NS-segment, it was shown that the PB2-segment tolerates the integration of foreign genes downstream of the PB2 ORF^11^,^12^,^46^. Since this approach represents an alternative to the NS segment as a vector for transgene expression, we generated pHW2000-based rescue plasmids encoding GFP or GLuc separated from the PB2 ORF by a PTV-1 2A-coding sequence (Fig. 7A). To guarantee packaging of the modified segment into viral particles, a terminal stretch of the segment was duplicated and fused downstream to the reporter gene as described by others^47^. Both viruses, encoding GFP (SC35M_PB2_2A_GFP_) or GLuc (SC35M_PB2_2A_GLuc_) could be successfully generated. Specific fluorescent signals were observed upon infection of A549 cells with SC35M_PB2_2A_GFP_ by live cell imaging microscopy (Fig. 7A) and significant levels of luciferase could be detected in the supernatant of MDCK-II cells infected with SC35M_PB2_2A_GLuc_ (Fig. 7B). Analysis of the replication efficiency of these viruses in MDCK-II cells, revealed a substantial attenuation of SC35M_PB2_2A_GLuc_ compared to SC35M (Fig. 7A). In sharp contrast, viral growth of SC35M_PB2_2A_GFP_ was, to our surprise, as efficient as wild type virus (Fig. 7A). However, analysis of viral plaques obtained with SC35M_PB2_2A_GFP_ revealed that only 1 out of 123 plaques was GFP positive (Tab. 1), indicating that the vast majority of these viruses lost the intact reporter gene after a single passage on human cells. Indeed, sequencing of the RNA extracted from six GFP-negative plaques revealed that the GFP gene was deleted, most likely by homologous recombination at the duplicated packaging signals. Interestingly, SC35M_PB2_2A_GLuc_ did not lose its reporter activity after 4 passages (Tab. 1), suggesting that smaller genes might be tolerated on the expense of viral fitness.

To improve the genetic stability of the GFP-encoding PB2 segment, the highest possible number of silent mutations was introduced into the last 129 nucleotides of the PB2 ORF to prevent homologous recombination at the duplicated packaging signals (Fig. 7C). This modification resulted in a virus, designated SC35M_PB2mod_2A_GFP_, which was strongly attenuated in cell culture but genetically stable over four passages in A549 cells (Tab. 1). To rule out the possibility that this attenuation resulted from mutation of the PB2 ORF rather than from insertion of the transgene, we deleted the GFP-coding sequence from the PB2 segment (Fig. 7D). This virus (SC35M_PB2mod_) replicated in cell culture almost as efficiently as SC35M_WT_. In summary, this suggests that stable integration of a transgene into the PB2-segment of SC35M is possible but associated with severe attenuation.

## Discussion

Influenza A reporter viruses are important tools to study the biology of IAV and screening approaches. However, these viruses show in general impaired viral replication efficiencies, probably resulting in an unfavorable selective pressure towards loss of the reporter gene. Especially the latter may cause difficulties in the interpretation of the results observed with reporter viruses. In this study, we developed a strategy that allows the stable integration of reporter genes into the NS-segment of IAV. We achieved this by converting the NS segment into a single ORF encoding NS1, the respective gene of interest and NEP. The reporter gene was flanked by two sequences coding for the porcine Teschovirus-1 2A peptide (PTV-1 2A), thereby allowing co-translational separation of NS1, the reporter protein and NEP. The feasibility of this method was demonstrated by the successful generation of recombinant IAV, encoding a variety of fluorescent reporter proteins or catalytically active enzymes. Dependent on the gene inserted into the NS segment, we observed various degrees of attenuation. Most importantly, these genetically modified viruses displayed a high genetic stability over 4 passages in cell culture as well as over a single passage in mice and can be readily used for a broad range of *in vitro* and *in vivo* applications.

The levels of viral mRNA transcripts coding for NS1 or NEP in influenza A virus-infected cells is regulated by splicing, resulting in defined protein ratios of NEP and NS1. This is of special importance with respect to recent findings suggesting that the relative amount of NEP coordinates the intracellular timing of an infection and that an aberrant NS1 to NEP ratio results in inhibition of viral replication^23^. Intriguingly, the co-translational processing by PTV-1 2A seems rather inefficient, resulting in a substantial proportion of uncleaved polyprotein in cells infected with the recombinant viruses (Fig. 1C). As a consequence, the level of free NEP is comparable to that detected in cells infected with wild type virus preventing significant attenuation as exemplified best with the recombinant virus SC35M_NS1_2A_NEP_ harboring no additional reporter gene (Fig. 1C). Interestingly, cells infected with SC35M_NS1_2A_GFP_2A_NEP_ expressed high levels of a GFP-2A-NEP fusion protein, while NS1-2A-GFP was almost not detectable (Fig. 3C and Fig. S1). This might be the result of context-specific differences in 2A-mediated recoding efficiency or could be explained by a higher stability of GFP-2A-NEP compared to NS1-2A-GFP.

Integration of foreign genes into the NS segment using the method presented in this study, can lead to impaired viral fitness. This might be a result of (I) low levels of NEP due to inefficient PTV-1 2A activity (Fig. 3C), (II) an intrinsic feature of the inserted reporter gene or (III) increased overall length of the NS segment. Indeed, integration of larger genes into the viral genome including firefly luciferase (~2 kb) or β-galactosidase (~3 kb) failed, suggesting a particular length restriction.

We could show for SC35M that insertion of the GFP gene into the PB2-segment results in the rapid loss of reporter activity due to homologous recombination of the duplicated packaging sequences (Fig. 7A and Tab.1). Loss of the reporter gene could be prevented by extensive modification of these sequences but was associated with significantly impaired viral growth (Fig. 7C and Tab.1). In contrast to the NS segment, introduction of reporter genes into the PB2 segment of SC35M did not result in viruses that are suited for further *in vitro* and *in vivo* studies. However, we cannot exclude the possibility that this might be different for other influenza A virus strains.

Importantly, the recombinant viruses described in this study harboring reporter genes in the NS segment are genetically stable in cell culture and mice. This includes fluorescent protein-encoding genes shown to be readily eliminated in cell culture or mice when present as NS1-fusion genes^15^,^16^,^20^. Genetically stable reporter viruses offer several advantages, including the reliable identification and determination of the relative abundance of virus-infected cells by microscopic tracing of these cells in a model organism. Because of their genetic stability, luciferase-encoding viruses might be robust tools for high throughput screening approaches. Here, SC35M_NS1_2A_RenLuc_2A_NEP_ might be superior, as the activity of the virus-encoded RenLuc allows measurements already early upon infection and parallels with viral titers at later time points (Fig. 4C).

Sublethal infection of mice with influenza A virus is cleared by day 10–14 and no infectious particles can be isolated from the lung of these animals there upon^48^. Using the Cre recombinase-encoding virus, we could trace cells that survived viral infection in the lungs of infected rosa^mT/mG^ mice 21 days after viral challenge. GFP-positive cells were found in various lung tissues including the epithelial layers lining the larger and also smaller airways (alveoli and bronchioles) of the respiratory tract. The fact that we could only observe the formation of GFP-positive cell clusters at later time points post infection (Fig. 5D) suggests that a proportion of these cells represents progeny of formerly infected and surviving cells, as GFP expression is inherited. Consistently, flow cytometry revealed the presence of GFP-positive cells among the stem and/or progenitor population 2, 7 and also 21 days post infection (Fig. 6B). Intriguingly, similar experiments were recently performed with an engineered H1N1 virus (A/Puerto Rico/8/1934) encoding a Cre recombinase within the PB2 segment^46^. In this case the authors could identify recombined cells exclusively in the larger airways of the respiratory tract. The reason for this discrepancy is unclear but could be related to differences in the segment used as vector for Cre recombinase-expression or might result from the different subtype of hemagglutinin (HA) of both mouse-adapted virus strains. While HA of PR/8 (H1N1) possesses a monobasic cleavage site, HA of SC35M (H7N7) harbors a multibasic cleavage site, which might facilitate infection of a broader spectrum of lung cells. Of note, influenza virus replication is known to be accompanied by the production of defective interfering particles (DIs) as well as semi-infectious particles (SIs)^49^,^50^,^51^,^52^. We cannot exclude the possibility that recombination events observed in cells of infected rosa^mT/mG^ mice were caused by the incorporation of such particles, which might not have the capacity to induce cell death. Importantly, the *in vivo* prevalence of DIs and SIs remains to be elucidated^53^.

Taken together, we present a strategy that permits the introduction of foreign genes into the NS genome segment of Influenza A viruses. This was achieved by conversion of the intron-containing NS segment into a segment that allows the expression of reporter genes by two porcine Teschovirus-1 2A peptide (PTV-1 2A)-coding sequences. Such engineered viruses were shown to be genetically stable over at least 4 passages in cell culture and a single passage in mice and thus represent attractive tools to study influenza A viruses *in vitro* and *in vivo.*

## Methods

### Plasmid construction

The Azurite gene was amplified from pGEMHE-X-Azurite, which was kindly provided by Max Ulbrich. The template used to amplify the Cre recombinase gene (pMIG-Cre) was a gift from Hassan Jumaa. The Gaussia Luciferase gene sequences derived from pT7-NYMVmg-Gluc^54^ and the Renilla luciferase-coding sequence was obtained from pRL-SV40 (Promega). The eGFP gene was amplified from pCAGGS-GFP-P^27^. pHW2000-based^26^ rescue plasmids to generate SC35M were described by others^32^. The NS1_2A_NEP plasmid was generated by deletion of the Flag-tag sequence of NS1_2A_Flag-NEP^24^ by performing overlapping fusion PCR. The same plasmid served as template to create NS1_2A_GFP-NEP where the Flag-tag sequence was replaced by an eGFP gene. NS1_2A_GFP_2A_NEP was obtained by insertion of a second genetically altered PTV-1 2A encoding sequence (GCCACAAATT TCTCTCTCCT CAAGCAAGCC GGGGACGTCG AGGAGAATCC CGGGCCC) between the genes for eGFP and NEP by overlapping fusion PCR. Furthermore, a SacII and a KpnI restriction site upstream and downstream of the eGFP gene respectively were introduced. NS1_2A_Renilla_2A_NEP and NS1_2A_Cre_2A_NEP were generated by overlapping fusion PCR using NS1_2A_GFP_2A_NEP as template. All other transgene-encoding NS-segment rescue plasmids were generated by PCR amplification of the transgene and SacII/KpnI-digestion-ligation into NS1_2A_GFP_2A_NEP. The PB2_2A_GFP and PB2_2A_GLuc plasmids were generated in two steps. First, a PB2_2A_GFP_2A_NEP and a PB2_2A_GLuc_2A_NEP intermediate was generated by PCR amplification of PB2 and digestion-ligation into NS1_2A_GFP_2A_NEP and NS1_2A_GLuc_2A_NEP. In a second step, the 2A_NEP sequence was replaced by 166 nucleotides of the 3’ end of the PB2 segment by overlapping fusion PCR. To obtain PB2mod_2A_GFP, silent mutations within the last 129 nucleotides of the 3’ end of the PB2 ORF (GCcAAaGGcG AaAAaGCcAA cGTcCTgATc GGcCAgGGcG AtGTcGTccT GGTcATGAAa aGaAaaGaGA tagctccATc CTgACcGAtt ccCAaACaGC cACaAAgAGg ATcaGaATGG CtATtAAc) were introduced into PB2_2A_GFP by annealing of two synthetic DNA oligonucleotides and subsequent overlapping fusion PCR. PB2mod was generated by removal of the 2A_GFP sequence from PB2mod_2A_GFP via PCR amplification and digestion-ligation.

### Cells and retroviral transduction

HEK293T, A549, Calu-3 and MDCK-II cells were grown in Dulbecco’s modified Eagle’s medium supplemented with 10% fetal calf serum, 2 mM L-glutamine and 1% penicillin/streptomycin. All cells were cultured at 37 °C and 5% CO_2_. Calu-3 cells harboring the loxp-dsRed-loxp-eGFP expression cassette were generated by retroviral transduction and subsequent selection in puromycine-containing media as described by the manufacturer (Stratgene). Pseudotyped retroviurses were produced in HEK293T cells upon transfection with pMSCV-loxp-dsRed-loxp-eGFP-Puro-WPRE (Addgene plasmid 32702, kindly provided by Hans Clevers), pVPack-GP and pVPack-VSV-G as decribed (Stratagene).

### Virus rescue

To generate recombinant influenza viruses, HEK293T cells were transfected in a 6 well format with 8 bidirectional pHW2000 rescue plasmids^26^ encoding the respective viral genome segments (300 ng of plasmid DNA/segment). 24 hours post transfection, 200 μl of supernatant was transferred to MDCK-II cells (6 well format). Recombinant viruses were plaque-purified upon the observation of cytopathic effects.

### Determination of viral growth kinetics

MDCK-II cells were infected at an MOI of 0.001 and cultured in infection medium (Dulbecco’s modified Eagle medium supplemented with 0.2% bovine serum albumin [BSA], 2 mM l-glutamine, and 1% penicillin-streptomycin). At 12, 24, 36 and 48 hours post infection 150 μl of supernatant was collected and subjected to plaque assay for determination of virus titer.

### Passaging and analysis of reporter stability

For each passage of fluorescent and luminescent reporter viruses, A549 cells were infected at an MOI of 0.01 and cultured in infection medium (see preceding chapter). 48 hours post infection, supernatant was collected and virus titer was determined by plaque assay. After 4 passages, plaques induced by fluorescent reporter viruses were analyzed and counted using a fluorescent microscope. To determine the reporter expression of passaged, luciferase-encoding viruses, 10 plaques were randomly picked and used for infection of MDCK-II cells and 24 hours post infection luciferase activity was measured. SC35M_NS1_2A_Cre_2A_NEP_ was passaged on MDCK-II cells. After 4 passages and subsequent plaque assay, Calu-3 cells containing the loxp-dsRed-loxp-eGFP expression cassette were infected with plaque-purified viruses. After 3 hours, medium was replaced by Ribavirin-containing (100 μM) medium. Recombination events resulting in a switch from red to green fluorescence were analyzed with a fluorescent microscope.

### Determination of luciferase activity

Luciferase activity in whole cell lysates or in 5 μl of supernatant of MDCK-cells cultured in 6-well plates was determined using a luciferase assay system (Promega) according to the manufacturer’s instructions.

### Immunoblot analysis

Virus infected cells were incubated with lysis buffer (20 mM Tris, pH 7.5, 100 mM NaCl, 0.5 mM EDTA, 0.5% NP-40, 1% protease inhibitor mix G [Serva, Heidelberg, Germany], 1 mM dithiothreitol [DTT]) for 15 min on ice. After centrifugation at 13,000 rpm at 4 °C, supernatants were complemented with SDS page sample buffer^55^ and incubated at 95 °C. Proteins were separated in SDS-PAGE gels (15%), and transferred to nitrocellulose membranes. Antibodies for detection of NEP and NS1 were a gift from Thorsten Wolff and Christina Ehrhardt respectively. The commercial antibodies for detection of GFP and tubulin were purchased from Santa Cruz Biotechnology (GFP) and Sigma-Aldrich (tubulin).

### Animal experiments

All animal experiments were performed in accordance with the relevant guidelines (German animal protection law (TierSchG)) and approved by the welfare committees of the University of Freiburg, as well as the local authorities. Six-to-eight-week-old mice were anaesthetized with a mixture of ketamin (100 μg per gram body weight) and xylazine (5 μg per gram) administered intraperitoneally and inoculated intranasally with the indicated doses of viruses in 40 μl PBS containing 0.3% bovine serum albumin (BSA). Animals were sacrificed, if severe symptoms developed, or body weight loss approached 25% of the initial value. Lung homogenates were prepared using the FastPrep24 system (MP Biomedicals). Briefly, after addition of 800 μl of PBS containing 0.2% BSA, lungs were subjected to two rounds of mechanical treatment for 10 s each at 6.5 ms^−1^. Tissue debris was removed by low-speed centrifugation. The LD_50_ values were calculated based on the infectious dose (PFU). BALB/c mice were obtained from Janvier (Strasbourg). Rosa^mT/mG^ mice (*Gt(ROSA)26Sor*^*tm4**(ACTB-tdTomato,-EGFP)Luo***^) (Jackson laboratory) contain the two-color fluorescent rosa^mT/mG^ allele from which the cell membrane localized red fluorescent tdTomato is expressed. Upon Cre-Lox recombination directed by lox-P sites flanking the tdTomato gene, eGFP expression is induced.

### Tissue histology

At appropriate time points, mice were sacrificed and lungs were transcardially perfused with 0.9% NaCl prior to fixation in 4% paraformaldehyde (PFA) in 0.1 M phosphate buffer at 4 °C overnight. After washing in ddH_2_O the lungs were first transferred into a 15% sucrose solution in 0.1 M phosphate buffer (w/v) for 4 h and thereafter in a 30% sucrose solution in 0.1 M phosphate buffer (w/v) overnight (4 °C). For cryosection, lungs were embedded within Tissue-Tek O.C.T. compound, solidified on dry ice and cut to 15 μm thickness using a cryotome (Leica Microsystems, Germany). The sections were mounted onto gelatine-coated slides and dried at room temperature overnight in the dark. The slides were washed twice in phosphate buffer, DAPI (Invitrogen) stained at an end concentration of 300 nM in 0.1 M phosphate buffer for 7 min and again washed 3 times in 0.1 M phosphate buffer. Dried slides were embedded within IMMU-Mount™ (ThermoShandon), coverslipped and stored in the dark at 4 °C until further use.

### Isolation of murine distal lung cells

Mouse lungs were perfused with HBSS (Gibco) followed by instillation of dispase (BD Biosciences) into the lung through the trachea and incubation in dispase for 40 minutes as previously described^56^. Trachea and large airways were dissected and the remaining distal lung tissue was homogenized (GentleMACS, MACS Miltenyi Biotech) in DMEM/2.5% HEPES with 0.01% DNase (Serva) and filtered through 100 μm and 40 μm nylon filters. Cell suspensions were incubated with biotinylated rat anti-mouse CD45, CD16/32 and CD31 mAb (BD Biosciences) for 30 minutes at 37 °C followed by incubation with biotin-binding magnetic beads and magnetic separation to deplete leukocytes and endothelial cells prior to flow cytometric analysis.

### Flow cytometry

The following antibodies were used for flow cytometric analyses: CD326 (EpCam) APC-Cy7 (clone G8.8), CD24 PE-Cy7 (clone: M1/69), T1α/podoplanin APC (clone: 8.1.1.), CD31 Pacific Blue (clone 390), all Biolegend. CD45 V450 (clone 30-F11, BD Biosciences). Multicolor flow cytometry was performed with an LSR Fortessa® using DIVA software (BD Bioscience). For analytical measurements 0.5–1 × 10^6^ cells were freshly stained with fluorochrome-labeled antibodies for 15 minutes at 4 °C in MACS buffer. The stained cells were washed and fixed in 4% paraformaldehyde, and resuspended in MACS buffer.

### Fluorescence microscopy

Fluorescence images of cultured cells seeded in black, clear bottom 96 well microplates (Greiner) were acquired on a Zeiss Observer.Z1 inverted epifluorescence microscope (Carl Zeiss, Jena) equipped with an AxioCamMR3 camera using a 40x objective. Fluorescence microscopy of lung sections was performed on a Zeiss Axioplan 2 epifluorescence microscope (Carl Zeiss, Jena) equipped with an ApoTome optical sectioning module using a 10x objective. Images were recorded with an AxioCamMR camera (Carl Zeiss, Jena).

## Additional Information

**How to cite this article**: Reuther, P. *et al.* Generation of a variety of stable Influenza A reporter viruses by genetic engineering of the NS gene segment. *Sci. Rep.* **5**, 11346; doi: 10.1038/srep11346 (2015).

## Supplementary Material

Supplementary Information

## Figures and Tables

**Table 1:** 

**SC35M**	**Positive plaques after 4 passages in culture**	**after one passage in BALB/c mice**
NS1_2A_GFP_2A_NEP	39/39	100/100
NS1_2A_Azurite_2A_NEP	44/44	100/100
NS1_2A_dsRed_2A_NEP	41/41	100/100
NS1_2A_RenLuc_2A_NEP	10/10	n.d.
NS1_2A_GLuc_2A_NEP	10/10	n.d.
PB2_2A_GLuc	10/10	n.d.
PB2_2A_GFP	1/123^*^	n.d.
PB2_mod__2A_GFP	12/12	n.d.
NS1_2A_Cre_2A_NEP	10/10**	10/10

Table 1.  Genetic stability of reporter viruses in cell culture. To determine the stability of the reporter genes encoded by the indicated viruses, A549 cells were infected at an MOI of 0.01. After 4 subsequent serial passages in A549 cells, plaque assay was performed for further analysis. To measure the stability of reporter genes after a passage in mice, plaque assay was performed on lung homogenates 2 days post infection. For fluorescent reporter viruses, plaques were analyzed by fluorescent microscopy. For the luciferase encoding viruses individual plaques were isolated and used to infect MDCK cells to determine luciferase activity. For the Cre-encoding virus, plaques were transferred to Calu-3 cells containing the loxp-dsRed-loxp-eGFP expression cassette in the presence of ribavirin (100 μM). *already after one passage. **passaging performed on MDCK-II cells. n.d.: not determined.

**Table 2:** LD_50_ of fluorescent reporter viruses.

	**LD_50_ (PFU)**
NS1_2A_GFP_2A_NEP	4 × 10^5^
NS1_2A_Azurite_2A_NEP	5 × 10^4^
NS1_2A_dsRed_2A_NEP	5 × 10^5^
SC35M	6 × 10^2^

LD50 of the indicated viruses after intranasal infection of BALB/c mice (n = 5 mice per group/ per infection dose). The LD_50_ of SC35M (10^28^ PFU) was determined elsewhere^32^.
