# Supplementary material for: Generation of a variety of stable Influenza A reporter viruses by genetic engineering of the NS gene segment
Source: Sci Rep. 2015 Jun 12;5:11346. doi: 10.1038/srep11346 (PMC4464305; doi:10.1038/srep11346)
Supplement: Supplementary Information [file srep11346-s1.pdf]

Supplementary information

Generation of a variety of stable Influenza A reporter viruses by genetic engineering of the NS gene segment

Peter Reuther[1,2,§], Kristina Göpfert[1,§], Alexandra H. Dudek[1,2,3], Monika Heiner[4], Susanne Herold[4] and Martin Schwemmle[1,*]

[1]Institute for Virology, [2]Faculty of Biology, [3]Spemann Graduate School of Biology and Medicine (SGBM), University of Freiburg, 79104 Freiburg, Germany and [4]Department of Internal Medicine (Pulmonology), University of Giessen and Marburg Lung Center, German Center for Lung Research, D-35392 Giessen, Germany

[§]These authors contributed equally to this work

*****Correspondence to:

Dr. Martin Schwemmle

Institute for Virology

Department of Medical Microbiology and Hygiene

University of Freiburg

Hermann-Herder-Strasse 11

D-79104 Freiburg, Germany

Phone: +49-761-203-6526

Fax: +49-761-203-6639

e-mail: martin.schwemmle@uniklinik-freiburg.de

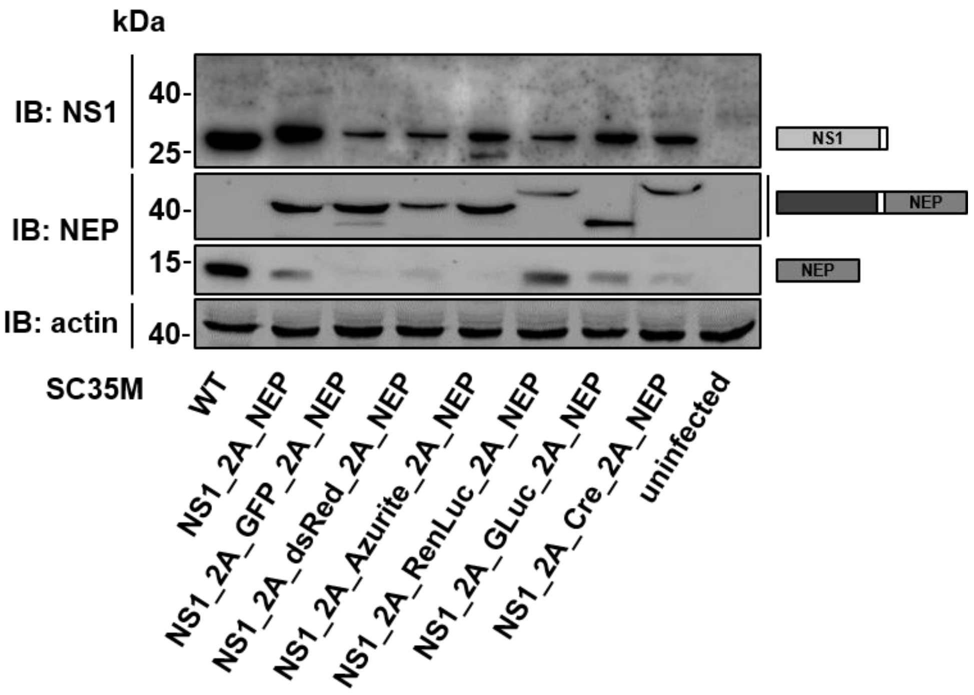

Fig.S1 NS1 and NEP protein expression levels of SC35M reporter viruses. Determination of expression levels of NS1 and NEP in A549 cells infected with $SC35M_{WT}$, $SC35M_{NS1\_2A\_NEP}$ and the respective reporter viruses. Cartoons indicate the approximate size of NS1_2A, NEP-fusion proteins and NEP. Approximate molecular weight of NS1: 25kDa; NEP: 14kDa; GFP, dsRed, Azurite: 27kDa; Renilla-Luciferase: 36kDa; Gaussia-Luciferase: 20kDa, Cre-recombinase: 38kDa.
